# The correlation between the European System for Cardiac Operative Risk Evaluation and the Model for End-Stage Liver Disease in patients with coronary artery bypass graft surgery

**DOI:** 10.25122/jml-2024-0311

**Published:** 2024-10

**Authors:** Andreea Ludusanu, Bogdan-Mihnea Ciuntu, Adelina Tanevski, Valentin Bernic, Grigore Tinica

**Affiliations:** 1 Department of Morpho-Functional Sciences I, Faculty of Medicine, Grigore T. Popa University of Medicine and Pharmacy, Iasi, Romania; 2 Department of General Surgery, Faculty of Medicine, Grigore T. Popa University of Medicine and Pharmacy, Iasi, Romania; 3 General Surgery Clinic, St. Spiridon County Emergency Clinical Hospital, Iasi, Romania; 4 Institute of Cardiovascular Diseases Prof. Dr. George I.M. Georgescu, Iasi, Romania; 5 Department of Cardiac Surgery, Faculty of Medicine, Grigore T. Popa University of Medicine and Pharmacy, Iasi, Romania

**Keywords:** EuroSCORE, MELD score, retrospective study, coronary artery bypass grafting surgery, risk assessment, liver disease

## Abstract

The Model for End-Stage Liver Disease (MELD) score is a widely used tool for quantifying hepatic dysfunction, providing greater accuracy and a wider range of values compared to the Child-Turcotte-Pugh (CTP) score, being also used in prioritizing patients who are eligible for liver transplantation. This study assessed the correlation between the MELD score and the European System for Cardiac Operative Risk Evaluation II (EuroSCORE II), a reliable system for categorizing risk levels in patients undergoing cardiovascular surgery. This retrospective study analyzed data from 589 patients who underwent coronary artery bypass grafting (CABG) at the Institute of Cardiovascular Diseases 'Prof. Dr. George I.M. Georgescu' in Iași between January 2011 and December 2020. Data collected included demographical, clinical, biochemical, and intraoperative parameters. The average MELD score was 6.09 ± 4.1 (median = 5.72), and the average EuroSCORE II was 6.28 ± 8 (median = 3.85). A significant but relatively modest positive relationship was found between the MELD score and EuroSCORE II, with a correlation coefficient of 0.23 and a corresponding significance level of 0.001. This study demonstrates a positive correlation between MELD and EuroSCORE II in patients who underwent CABG. Incorporating the MELD score into the preoperative risk assessment of cardiac surgery patients could help identify high-risk individuals and guide clinical decision-making.

## INTRODUCTION

Coronary artery disease, also known as CAD, is a chronic disease characterized by plaque accumulation in the arteries that supply the heart with oxygen-rich blood [1]. This plaque accumulation, known as atherosclerosis, can lead to the narrowing and hardening of the arteries, ultimately reducing blood supply to the cardiac tissue [2]. Despite recent progress in comprehending and managing CAD, it continues to be the leading cause of death worldwide, with over 900,000 persons only in the United States of America (USA) experiencing a myocardial infarction or dying from the condition annually [3]. The pathogenesis of coronary artery disease is a complex and multifaceted process. It is believed to begin with endothelial dysfunction, where the cells lining the coronary arteries can no longer effectively regulate vascular tone with nitric oxide signaling [4,5]. This malfunction enables the gradual invasion of the blood vessel wall by cholesterol-bearing lipoprotein particles, prompting an inflammatory reaction from cholesterol-engorged macrophages, also named the 'foam cells' [5]. Underlying nonstriated muscle cell proliferation leads to vessel remodeling, ultimately narrowing the lumen and restricting blood flow to the heart muscle [4-6]. The severity of the condition is determined by the specific coronary arteries affected and their quantity, with treatment options chosen accordingly. In less severe cases, percutaneous coronary intervention, or coronary angioplasty, is a widely accepted less invasive treatment approach [4,7]. In more severe cases, bypass surgery can reroute blood flow around the blocked coronary arteries [6,7].

Coronary artery bypass grafting (CABG) is an effective revascularization technique; however, concerns persist regarding the associated mortality and surgical complications in patients with stable angina pectoris [8]. Several preoperative risk assessment systems have been developed to predict patient outcomes, utilizing angiography and laboratory test results. Among the most widely used tools are the European System for Cardiac Operative Risk Evaluation II (EuroSCORE II), the Age, Creatinine, and Ejection Fraction Score (ACEF Score), and the Society of Thoracic Surgeons short-term risk score (STS Score), which considers a patient's age, creatinine levels, and ejection fraction [9].

The Model for End-Stage Liver Disease (MELD) was initially designed to forecast the outcomes of individuals with chronic liver disease [10]. Prior research has identified liver disease as a negative prognostic factor for cardiac surgery patients, while current operative risk assessment tools such as STS and EuroSCORE II do not incorporate precise metrics for assessing liver dysfunction [11]. Several studies have established an association between an elevated MELD score and increased long-term mortality among patients with acute coronary syndromes who undergo percutaneous coronary angioplasty [12,13]. Individuals with end-stage liver disease who undergo non-transplant surgical procedures experience significantly higher mortality and surgical complication rates compared to those without liver disease [13,14]. Various clinical factors associated with the degree of liver dysfunction, including the Child-Turcotte-Pugh (CTP) and MELD scores, have been shown to reliably predict postoperative outcomes [14]. This study aimed to assess the relationship between the EuroSCORE II and the MELD score in predicting adverse health outcomes, including mortality, in patients undergoing coronary artery bypass graft surgery.

## MATERIAL AND METHODS

### Patient selection

This was a retrospective observational study that included patients diagnosed with coronary artery disease who underwent coronary artery bypass grafting procedures at our institution between January 1st, 2011, and December 31st, 2020. Data was collected from electronic medical records, including biological information at admission and discharge, imaging findings, and details of surgical procedures. To protect patient privacy, each participant was assigned a unique identifier, and the study results were used in compliance with a protocol approved by the Ethics Committee, number 163/21.03.2022. The study analyzed a cohort of patients who received CABG and had complete medical records, excluding patients with coronary artery disease who underwent percutaneous angioplasty and those with incomplete medical records.

### Statistical analysis

The study data was analyzed using IBM SPSS Statistics 25 and presented using Microsoft Office Excel/Word 2021. Quantitative variables were assessed for normal distribution through the Shapiro-Wilk Test and Pearson correlation coefficient and reported as means with standard deviations or medians with interquartile ranges, as appropriate. Non-parametric variables were compared between groups using the Mann–Whitney U test. Qualitative variables were summarized as counts or percentages and compared between groups employing Fisher's exact test. All statistical analyses were performed at a significance level of 0.05 [15].

### Variables analyzed

The study extracted variables necessary for calculating the MELD score and EuroSCORE II, as well as other factors critical for assessing patient health status, including:

- Demographic parameters: age, sex.
- Biological assessment: complete blood count (CBC), coagulation parameters, and ionogram (electrolyte) tests at admission and discharge.
- Echocardiography data: ejection fraction (%), types of valve insufficiency, and degree of pulmonary hypertension (medium/severe).
- Intra-operative protocol data: height (cm), weight (kg), type of surgery (simple CABG, other type of cardiac surgery except CABG, multiple surgical procedures), surgical regimen (emergency, elective, major emergency, salvage), and thoracic aortic surgery.
- Data from clinical observation sheet: personal pathologic history (chronic pulmonary disease, extracardiac arteriopathy, musculoskeletal or neurologic disorders), previous surgery involving pericardial closure, active endocarditis on admission, critical preoperative status (ventricular tachycardia, ventricular fibrillation, preoperative resuscitated cardiac arrest, preoperative presence of intra-aortic balloon pump or acute renal insufficiency), insulin-dependent diabetes mellitus, class IV angina pectoris, acute myocardial infarction (within the last 90 days), and chronic heart failure, classified according to the New York Heart Association (NYHA) functional classification.

Liver function was assessed using the MELD score, calculated upon admission using the official formula:

MELD Score = 3.78 × ln(bilirubin) + 11.2 × ln(creatinine) + 9.57 × ln + 6.43 [16].

The MELD score was then interpreted based on the values in Table 1.

**Table 1:** MELD score interpretation

3-month mortality, %	MELD score
1.9-3.7	<9
6-20	10-19
19.6-45.5	20-29
52.6-74.5	30-39
71-100	>40

MELD, model for end-stage liver disease

A class was associated with each mortality interval to streamline the statistical process (Table 2).

**Table 2:** Interpretation of MELD score and class

MELD Score	3-month mortality (%)	MELD class
< 9	1.9%-3.7%	1
10 to 19	6%-20%	2
20 to 29	19.6%-45.5%	3
30 to 39	52.6%-74.5%	4
>40	71%-100%	5

**Figure 1:**
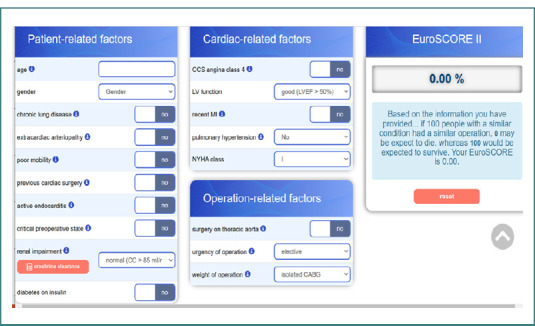
Official EuroSCORE II calculator (source: https://www.euroscore.org/calc.html).

To assess the risk of mortality following surgery, EuroSCORE II was calculated for all patients using the official calculator available on the EuroSCORE II website (Figure 1). This tool computes the mortality risk automatically based on the entered patient-specific variables, providing a standardized and reliable measure of operative risk.

## RESULTS

A total of 589 patients who underwent CABG with cardiopulmonary bypass (CPB) during the study period were included in the analysis. The baseline characteristics and admission parameters are summarized in Table 3. The mean age of patients was 63.1 ± 8.98 years (median = 64 years), with a majority of male participants (78.6%), Rh positive (85.4%), with blood types A (46.4%) or O (29.4%). The mean length of hospitalization was 19.19 ± 7.5 days (median = 17). The rest of the parameters aligned with the clinical profiles of CAD patients characterized by a high prevalence of valvular insufficiency and atypical ECG and echocardiography findings.

**Table 3:** Baseline characteristics and biochemical parameters of study participants at admission

Parameters	Value
**Age (Mean ± SD, Median (IQR))**	63.1 ± 8.98, 64 (58-70)
**Blood Group (No., %)**	172 (29.4%) O, 272 (46.4%) A, 96 (16.4%) B, 46 (7.8%) AB
**Rh Positive (No., %)**	503 (85.4%)
**Sex (No., %)**	126 (21.4%) F, 463 (78.6%) M
**Length of hospitalization**	19.19 ± 7.5, 17 (15-22)
***Serum biochemistry at admission (Mean ± SD, Median (IQR))***	
Thrombocytes (*1000)	230.29 ± 62.18, 223 (192-264)
**Leukocytes**	8136.6 ± 4981.7, 7600(6552-8980)
**% Lymphocytes**	27.24 ± 8.88, 26.4 (21.4-32.3)
**% Polymorphonuclear**	61.96 ± 9.72, 62.9 (56.2-68.7)
**% Monocytes**	7.73 ± 3.27, 7.2 (6.1-8.72)
**% Eosinophils**	2.52 ± 2.56, 1.9 (1.2-3.1)
**Hematocrit**	40.52 ± 4.26, 40.5 (38.04-43.3)
**Hemoglobin**	13.68 ± 1.41, 13.75 (12.82-14.63)
**The sedimentation rate of hematite**	22.51 ± 15.24, 20 (10-32)
**Prothrombin quick time**	15.04 ± 2.37, 14.7 (13.9-15.5)
**International normalized ratio**	1.08 ± 0.24, 1.03 (0.97-1.12)
**Time of partially activated thromboplastin**	30.43 ± 7.28, 29.1 (26.6-32.6)
**Prothrombin Index**	93.34 ± 19.36, 94 (83-105)
**Fibrinogen**	479.96 ± 130.19, 447 (388-576)
**Natrium**	139.36 ± 3.43, 140 (138-141)
**Potassium**	4.54 ± 1.81, 4.5 (4.2-4.7)
**Blood Glucose**	125.56 ± 44.19, 113 (99-138)
**Urea**	41.32 ± 16.17, 38 (31-48)
**Creatinine**	1.05 ± 0.61, 0.96 (0.86-1.12)
**Bilirubin**	0.84 ± 0.39, 0.78 (0.56-1.03)
**AST-Aspartate Aminotransferase**	29.9 ± 18.68, 24.5 (19.25-34)
**ALT-Alanine Aminotransferase**	40.12 ± 32.76, 29.5 (21-47)
**Triglycerides**	142.56 ± 79.31, 122 (92-173.5)
**Total Cholesterol**	168.41 ± 47.9, 161 (135-192)
***Echocardiographic parameters – Admission (Mean ± SD, Median (IQR))***	
TS segment	99.33 ± 4.96, 100 (97.5-102)
PR segment	0.172 ± 0.034, 0.17 (0.16-0.18)
QRS complex	0.108 ± 0.104, 0.1 (0.08-0.1)
RS segment	71.03 ± 11.25, 70 (63-79.25)
QT segment	0.398 ± 0.023, 0.4 (0.39-0.4)
QRS axis	18.48 ± 37.27, 20 (0-45)
**Radiological examination-**Cardio-Thoracic Index-CTR	0.475 ± 0.06, 0.48 (0.44-0.51)
**Preoperative echocardiography**	
FS (%)-Fractional Shortening	28.55 ± 5.55, 30 (25-30)
EF (%)-Ejection Fraction	54.54 ± 8.56, 60 (50-60)
EF vol%-Volumetric Ejection Fraction	49.08 ± 11.27, 50 (40-59)
End-Diastolic Diameter of the Left Ventricle (mm)	40.92 ± 19.04, 50 (35-51)
End-Systolic Diameter of the Left Ventricle (mm)	32.67 ± 4.88, 33 (29.5-35.5)
TAPSE-Tricuspid Annular Plane Systolic Excursion	24.33 ± 13.97, 24 (21-26)
Mitral Valve Orifice Area (cm^2^)	3.74 ± 1.34, 3.6 (3.1-4.2)
Maximum Gradient Across the Tricuspid Valve	26.05 ± 10.89, 25 (18-32)
***Incidence of valve insufficiency-admission (No., %)***	
***Mitral insufficiency***	
**Absent**	120 (20.4%)
**Grade I**	358 (60.8%)
**Grade II**	80 (13.6%)
**Grade III**	27 (4.6%)
**Grade IV**	4 (0.7%)
***Aortic insufficiency***	
**Absent**	411 (69.8%)
**Grade I**	129 (21.9%)
**Grade II**	35 (5.9%)
**Grade III**	10 (1.7%)
**Grade IV**	4 (0.7%)
**Pulmonary insufficiency**	
**Absent**	541 (91.9%)
**Grade I**	42 (7.1%)
**Grad II**	6 (1%)
**Tricuspid insufficiency**	
**Absent**	171 (29%)
**Grade I**	381 (64.7%)
**Grade II**	34 (5.8%)
**Grade III**	3 (0.5%)

Table 4 describes the intra-operative parameters. The mean aortic clamping time was 88.19 ± 48.54 minutes (median = 82), and the total CPB duration was 118.45 ± 41.64 minutes (median = 112). The mean height was 169.52 ± 8.66 (median = 170), the mean weight was 84.73 ± 15.73 (median = 84), and the mean nadir temperature during the CPB was 33.8 ± 0.83 (median = 34). The mean ICU stay post-surgery was 7.27 ± 4.21 days (median = 6).

**Table 4:** Description of intra-operative parameters

Parameter (Mean ± SD, Median [IQR])	Value
Cross-clamp time (minutes)	88.19 ± 48.54, 82 (67-102)
Duration of CPB (minutes)	118.45 ± 41.64, 112 (95.75-135.25)
Height	169.52 ± 8.66, 170 (165-175)
Weight	84.73 ± 15.73, 84 (75-94)
Nadir temperature (°C)	33.8 ± 0.83, 34 (34-34)
Length of stay in ICU (days)	7.27 ± 4.21, 6 (5-8)

Table 5 illustrates the distribution of patients across MELD classes at admission. The majority of patients (87.3%) were classified as Class 1, followed by 11.4% in Class 2, 1.0% in Class 3, and 0.3% in Class 4. These results indicate that most patients had mild liver dysfunction, with only a small proportion experiencing more severe impairment.

**Table 5:** Frequencies of MELD classes at admission

MELD class		Rate	Percentage
Valid	Class 1	514	87,3
	Class 2	67	11,4
	Class 3	7	1,0
	Class 4	2	0,3
	Total	589	100

As shown in Table 6, the mean MELD score at admission was 6.09 ± 4.1 (median = 5.72), which increased to 9.2 ± 6.32 (median = 7.44) at discharge. The mean EuroSCORE II was 6.28 ± 8 (median = 3.85).

**Table 6:** Comparative analysis of MELD score and EuroSCORE II in cardiac surgery risk assessment

Parameter (Mean ± SD, Median [IQR])	Value
MELD score - admission	6.09 ± 4.1, 5.72 (3.55-8.21)
MELD score – discharge	9.2 ± 6.32, 7.44 (5.13-11.71)
EuroSCORE II	6.28 ± 8, 3.85 (2.3-6.98)

The data in Table 7 compares MELD scores at admission by sex. The score distribution was non-normal in both groups based on the Shapiro-Wilk test. The statistical analysis using the Mann-Whitney U test revealed significant differences between the groups, with men in the study demonstrating a significantly higher MELD score (median = 6.19, IQR = 4.27-8.48) compared to women (median = 3.66, IQR = 2.12-6.26).

**Table 7:** Comparison of admission MELD scores between male and female participants

Sex	Mean ± SD	Median (IQR)	Mean rank	*P* ^*^
Women (*P* < 0.001^**^)	4.23 ± 4.14	3.66 (2.12-6.26)	208.06	<0.001
Men (*P* < 0.001^**^)	6.6 ± 3.95	6.19 (4.27-8.48)	318.66	

*Mann-Whitney U Test, **Shapiro-Wilk Test

The correlation between the MELD score at admission and the length of hospitalization is presented in Table 8. According to the Shapiro-Wilk test (*P* < 0.05), both variables exhibited non-parametric distribution. A significant but weak positive correlation was observed (*P* = 0.009, R = 0.108), indicating that higher MELD scores were associated with longer hospital stays and vice versa.

**Table 8:** Correlation between MELD score at admission and length of hospitalization

Variable	Correlation coefficient (r)	*P*
MELD score × Hospitalization period	r = 0.108	0.009,

The bivariate Pearson correlation was calculated between each pair of variables. Table 9 summarizes the results, highlighting a weak but statistically significant positive correlation between the EuroSCORE II and MELD Score (r = 0.23, *P* < 0.001). Figure 2 illustrates this relationship, with the regression line showing an upward trend and a cluster of dotted values on the right, indicating a positive association. This suggests that an increase in one score is generally accompanied by an increase in the other.

**Figure 2:**
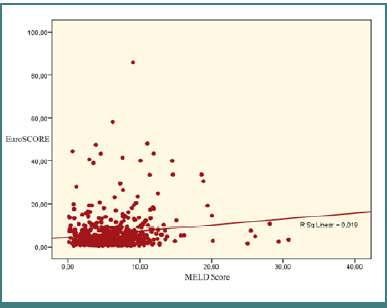
The regression line for EuroSCORE II and MELD score

**Table 9:** Bivariate Pearson correlation coefficient between EuroSCORE II and MELD score

Variables Compared	*n*	Correlation coefficient (r)	*P*
EuroSCORE II & MELD Score	589	0.23	<0.001

As shown in Table 10, the majority of patients at discharge had good global ventricular function, with an average ejection fraction of 51.46 ± 8.16%. Mitral and tricuspid valve insufficiencies were still present, with the mitral valve being the most commonly affected. Regarding serum biochemistry, some parameters, including thrombocytes, aspartate aminotransferase (AST), alanine aminotransferase (ALT), and urea, remained elevated or showed further increases compared to admission levels.

**Table 10:** Characteristics of the sample analyzed in the study with the parameters at discharge

Parameters	Value
***Parameters-Discharge (Mean ± SD, Median (IQR))***	
Thrombocytes (^*^1000)	379.85 ± 150.43, 368 (267.7-470.7)
**Leukocytes**	10133.5 ± 8424, 9400 (7600-11230)
**% Lymphocytes**	23.1 ± 8.67, 22.7 (17-28.2)
**% Polymorphonuclear**	64.23 ± 9.68, 64.6 (58-70.5)
**% Monocytes**	9.15 ± 3.21, 8.6 (7.1-10.62)
**% Eosinophils**	3 ± 1.83, 2.7 (1.8-3.8)
**Hematocrit**	33.81 ± 4.01, 33.82 (30.93-36.59)
**Hemoglobin**	11.35 ± 1.47, 11.38 (10.32-12.34)
**The sedimentation rate of hematite**	78.74 ± 30.07, 78 (56.75-100.5)
**Prothrombin Quick Time**	21.22 ± 10.87, 16.2 (15.1-23.72)
**International Normalized Ratio**	1.51 ± 0.83, 1.15 (1.06-1.41)
**Time of Partially Activated Thromboplastin**	34.83 ± 11.54, 32.25 (28.85-37.75)
**Prothrombin Index**	68.78 ± 28.23, 77 (44.5-89)
**Fibrinogen**	766.44 ± 152.98, 774 (673-857)
**Natrium**	135.95 ± 6.23, 136 (134-138.57)
**Potassium**	4.44 ± 0.53, 4.45 (4.1-4.8)
**Blood glucose**	122.22 ± 39.4, 112 (97-138.75)
**Urea**	45.45 ± 21.29, 41 (33-53)
**Creatinine**	1.06 ± 0.41, 1.01 (0.86-1.17)
**Bilirubin**	0.85 ± 0.5, 0.75 (0.58-1)
**AST**	39.41 ± 61.65, 29 (21-43)
**ALT**	65.8 ± 117.39, 46.5 (31-74.5)
**Triglycerides**	177.3 ± 74.98, 163.5 (128.75-210)
**Total cholesterol**	175.44 ± 42.92, 172.5 (145.7-199.2)
***Discharge echocardiography***	
FS (%) - Fractional Shortening	27.72 ± 5.25, 25 (25-30)
EF (%) - Ejection Fraction	51.46 ± 8.16, 50 (50-60)
EF vol% - Volumetric Ejection Fraction	48.11 ± 9.93, 50 (40-55)
TAPSE - Tricuspid Annular Plane Systolic Excursion	16.77 ± 3.28, 17 (15-19)
Mitral Valve Orifice Area (cm^2^)	4.01 ± 0.92, 3.9 (3.4-4.5)
Maximum Gradient Across the Tricuspid Valve	22.45 ± 7.29, 21 (17-27)
***Incidence of valve insufficiency-discharge (No., %)***	
***Mitral insufficiency***	
**Absent**	267 (45.3%)
**Grade I**	299 (50.8%)
**Grade II**	23 (3.9%)
***Aortic insufficiency***	
**Absent**	526 (89.3%)
**Grade I**	55 (9.3%)
**Grade II**	8 (1.4%)
***Pulmonary insufficiency***	
**Absent**	588 (99.8%)
**Grade I**	42 (7.1%)
***Tricuspid insufficiency***	1 (0.2%)
**Absent**	309 (52.5%)
**Grade I**	265 (45%)
**Grade II**	14 (2.4%)
**Grade III**	1 (0.2%)

## DISCUSSION

The observed clinical and echocardiographic characteristics of the study participants are consistent with the expected profile of patients admitted with coronary artery disease, as supported by previous research [17,18]. The mean age of 63.1 ± 8.98, the predominance of men, and the prevalence of cardiovascular risk factors reflect the epidemiological features commonly associated with coronary artery disease. Similarly, the laboratory findings, including hematological, biochemical, and coagulation profiles, such as hypercholesterolemia and hypertriglyceridemia, correspond to the typical alterations observed in the acute phase of myocardial infarction and the chronic one [18-20].

Consistent with previous reports, patients with extended ICU stays were older, underwent more complex surgical procedures, and had higher rates of pre-existing medical conditions. [21]. Overall, the results show that patients had improvements in most parameters at discharge compared to admission, except for some liver and kidney function tests such as AST, ALT, urea, and creatinine, which worsened. Also, the MELD score at discharge was higher than at admission, reflecting a potential decline in liver function during hospitalization. Furthermore, many patients continued to experience valve insufficiencies at discharge, underscoring the importance of close monitoring and care for these individuals [22,23]. A recent population-based study revealed that intensive care unit survivors experienced the highest rates of death, readmission in the hospital, and medical costs within the first 6 months following discharge [23-25]. This highlights patients' challenges in the immediate post-ICU period and the need for continued support and monitoring during this vulnerable time [24].

The distribution of MELD score among patients undergoing bypass grafting surgery indicates that the majority had well-preserved liver function at admission, with 98.7% classified in MELD Classes 1 and 2. The MELD score was initially developed to prioritize liver transplant candidates based on their risk of mortality [25]. However, it has since been explored for its potential in risk stratification for other medical conditions, including cardiovascular diseases. [26]. Research focused on patients undergoing coronary artery bypass grafting found that both MELD and MELD-XI (a version excluding INR) scores were linked to overall mortality and complications, with threshold values of 8.1 and 14.4, respectively [19,26,27]. The reported scores indicated high sensitivity (92.3% and 65.6%) but moderate specificity (61.5% and 84.4%) [25,27]. In particular, MELD and MELD-XI scores could predict mortality related to cardiac dysfunctions in patients undergoing CABG procedures [28,29]. Pathare *et al*. demonstrated that the MELD score is especially effective in predicting outcomes for high-risk patients undergoing cardiac surgery with CPB [25,30-32].

In the present study, a statistically significant but modest correlation was observed between the MELD score and EuroSCORE II, suggesting that the MELD score may provide valuable insights into the risk profile of cardiac surgery patients, potentially complementing or even enhancing existing risk assessment tools.

### Limitation

Limitations of this study include the retrospective design and the potential for unmeasured confounding factors. This was a retrospective observational study conducted over 10 years. During this time, surgical techniques, cardiopulmonary bypass, and anesthesia underwent significant changes. Furthermore, the study was limited to a single-center experience with a relatively small sample size, which may restrict the generalizability of the findings. Despite these limitations, the study provides valuable insights and may serve as a foundation for future prospective studies exploring liver dysfunction in patients undergoing coronary artery bypass grafting surgery.

## CONCLUSION

The study revealed a modest yet positive association between the MELD score and EuroSCORE in patients receiving coronary artery bypass grafting. While the MELD score has been widely used as a predictor of liver disease outcomes, its applicability in the context of cardiac surgery has not been well-explored. The findings suggest that the MELD score may help assess risk in patients undergoing coronary artery bypass grafting, especially those with underlying liver conditions. Additional studies are necessary to confirm these results and investigate the possible consequences for patient care and clinical outcomes. Integrating the MELD score into the preoperative risk evaluation of cardiac surgery patients may assist in identifying high-risk individuals and informing clinical decision-making, potentially enhancing outcomes for this vulnerable patient group.
